# A scoping review of research on complementary and alternative medicine (CAM) and the mass media: Looking back, moving forward

**DOI:** 10.1186/1472-6882-8-43

**Published:** 2008-07-19

**Authors:** Laura C Weeks, Tina Strudsholm

**Affiliations:** 1 Department of Community Health Sciences, University of Calgary, 3330 Hospital Drive NW, Calgary, AB, Canada

## Abstract

**Background:**

The use of complementary and alternative medicine (CAM) has become more common in Western developed countries in recent years, as has media reporting on CAM and related issues. Correspondingly, media reports are a primary information source regarding decisions to use CAM. Research on CAM related media reports is becoming increasingly relevant and important; however, identifying key concepts to guide future research is problematic due to the dispersed nature of completed research in this field. A scoping review was conducted to: 1) determine the amount, focus and nature of research on CAM and the mass media; and 2) summarize and disseminate related research results.

**Methods:**

The main phases were: 1) searching for relevant studies; 2) selecting studies based on pre-defined inclusion criteria; 3) extracting data; and 4) collating, summarizing and reporting the results.

**Results:**

Of 4,454 studies identified through various search strategies, 16 were relevant to our objectives and included in a final sample. CAM and media research has focused primarily on print media coverage of a range of CAM therapies, although only a few studies articulated differences within the range of therapies surveyed. Research has been developed through a variety of disciplinary perspectives, with a focus on representation research. The research reviewed suggests that journalists draw on a range of sources to prepare media reports, although most commonly they cite conventional (versus CAM) sources and personal anecdotes. The tone of media reports appears generally positive, which may be related to a lack of reporting on issues related to risk and safety. Finally, a variety of discourses within media representations of CAM are apparent that each appeal to a specific audience through resonance with their specific concerns.

**Conclusion:**

Research on CAM and the mass media spans multiple disciplines and strategies of inquiry; however, despite the diversity in approach, it is clear that issues related to production and reception of media content are in need of research attention. To address the varied issues in a comprehensive manner, future research needs to be collaborative, involving researchers across disciplines, journalists and CAM users.

## Background

At the same time as approximately one-half of the population in Western developed countries uses some form of complementary and alternative medicine (CAM) [1-4], CAM related media reports have become more common [5-7]. By their nature, media reports cannot be complete and are potentially biased and/or unbalanced; however, they are commonly used to support decisions related to CAM use [8,9]. CAM related media coverage and how audiences respond to such coverage is of great concern, as it appears that–not unlike conventional medical reporting–such information is insufficient to support informed decision-making [5,10].

Media research is commonly categorized into three main areas: production, representation and reception by audiences [11]. Research in each area can collectively contribute to an understanding of the complicated relationship between the media, society, culture, CAM use, and related beliefs and behaviour. For example, production research might examine the constraints that journalists face when producing CAM content or the relationship between journalists and their sources. Representation research may examine what is reported in the media about CAM and how often, as well as how CAM information is reported. Reception research may examine how audiences use media information and how that may impact their beliefs regarding CAM as well as their decision to use, or not use, CAM. Each area deserves research attention, as media information has clear potential to impact the decisions of patients, health care providers and policy makers, as well as the professionalization, legitimization and commodification of CAM.

Of course, all research related to CAM and the mass media needs to be theoretically informed, which includes a comprehensive knowledge of the current body of research literature. Identifying relevant research, however, is somewhat problematic as–by its nature–the field is multi-disciplinary, crossing the disciplines of health care, communication, sociology, cultural studies and others. Researchers tend to be comfortable with the literature in only one or a few of these disciplines but a narrow review focused on only a few disciplines is likely to exclude important evidence on this topic.

The purpose of a scoping review is to identify, retrieve and summarize literature relevant to a particular topic for the purpose of identifying the key concepts underpinning a research area and the main sources and types of evidence available [12]. Scoping reviews are similar to systematic reviews, but the objectives are broader and more comprehensive. Systematic reviews are narrow in focus and are guided by specific research questions [13-15], which is inappropriate for an assessment of the current status of research on CAM and the mass media.

A scoping review of research on CAM and the mass media was conducted with the objectives to: 1) describe the scope (i.e., amount, focus and nature) of research activity in this field; and 2) summarize and disseminate research results.

## Methods

Scoping reviews follow many of the same methodological steps as systematic reviews [13-15], as the use of rigorous and transparent methods for data collection, analysis and interpretation remains essential to enhance reliability of results and the potential for replication. A key difference between scoping and systematic reviews, however, is that quality assessments are not typical for scoping reviews [12] due to differing conceptions of what quality means [16]. In scoping reviews the focus is on the research findings themselves, as opposed to the means used to obtain them [17]. Therefore, the main phases of this scoping review were: 1) searching for relevant studies; 2) selecting studies based on pre-defined inclusion criteria; 3) extracting data; and 4) collating, summarizing and reporting the results. Although presented as a series of stages, the process was not linear but iterative. We moved flexibly through each stage, repeating steps when needed to ensure the literature was covered in a comprehensive way [12].

### Definitions and search strategies

We operationalized CAM as a set of examples from a core list of CAM products and therapies presented by the International Society for Complementary Medicine Research [18]. For example, CAM therapies in our search included acupuncture, chiropractic, homeopathy, meditation and massage therapy, among others. Some CAM products included were dietary supplements, vitamins and minerals (see CAM keywords or subject headings in Additional file 1).

We defined media as various communication channels capable of reaching broad and heterogeneous audiences; but, we limited our review to the traditional "mass" media of radio, television, newspapers and magazines. We excluded the Internet and other specialized media, primarily as a means to focus our review but also due to some fundamental differences between these media types. Audiences tend to engage with these media types in a more interactive, versus unidirectional, manner than traditional mass media [19]. Further, the nature of the production of traditional mass media content differs substantially from the production of Internet content. Traditional mass media content tends to be produced by a few multi-national corporations, while Internet content tends to be produced by a wider variety of sources. We focused on traditional mass media, as a means to bound the scope of our research, although we recognize the growing importance of Internet information for health care decision-making [20].

References to original research in the field of CAM and the traditional mass media were sought by searching electronic databases (health care, communication studies, sociology, social sciences), contacting authors and researchers in the field, distributing an email to over 150 members of the International Society for Complementary Medicine Research, and screening reference lists of identified articles [13,14,21].

For each database searched, we worked with a librarian from the primary discipline to develop a list of relevant keywords. In the case of some non-medically oriented databases, no CAM specific keywords were available and so we developed a set of terms applicable to general health care (see Additional file 1). To conduct the search, each CAM/health care keyword was combined with each media keyword listed in Additional file 1 using the Boolean operator 'AND', limiting the publication date to 1990 and later. Due to time and cost considerations, we limited our search to English language publications.

### Study selection

Of the studies identified through the various search strategies we used a standardized form to select those into our final sample that were relevant to our research objectives, guided by the following inclusion criteria: 1) media type of television, radio, newspapers or magazines; 2) CAM product or therapy on the ISCMR core list; and 3) English language publication. No exclusion criteria were defined based on study design or publication type [12], as long as the article described original research. Further and as is typical in scoping reviews, we did not use study quality as an inclusion criteria [12], although we did broadly assess indicators of study quality as a means to understand the nature of research methods used and reported in this diverse field [16].

The list of article titles resulting from the various searches was scanned by two reviewers, who each assigned a value of "include", "exclude" or "maybe" to each reference. In cases where it was impossible to make a decision based on the title alone, the full article was retrieved.

### Data extraction

We used a standard coding template to extract data from each original research article that would enable us to describe the amount, focus and nature (i.e. the scope) of research related to CAM and the mass media, as well as to summarize and disseminate the results of published research. To describe the amount of research in this field, we recorded the year of publication of each article. To describe the focus of the research, we extracted data regarding the media type, country of media origin as well as the disease and type of CAM product or therapy that were the subject of the research. To describe the nature of the research, we extracted data regarding the researchers' disciplinary background, the type of article, the research approach, characteristics related to methodological reporting and assessment, and the type of media research (i.e., production, representation, reception). In addition to extracting descriptive information, we extracted statements from each article that indicated original research results, being careful to distinguish results from the researchers' original data, results presented from different research studies in the form of discussion as well as researchers' discussion of their own findings [22].

Each author independently extracted the data from each article and entered them into an Excel database.

### Collating, summarizing and reporting the results

We used a qualitative descriptive approach [23] to summarize the results, grouping together statements we judged to be topically similar [24]. We produced both a descriptive summary of the research, using the categories of amount, focus and nature of the research, as well as a summary of research results. Given our objective to scope the field, our intent was to summarize the main results as presented across articles, not to synthesize or distil only those results that could help to answer a narrow research question [22].

## Results

### Search strategy, study selection and data extraction

**Figure 1:**
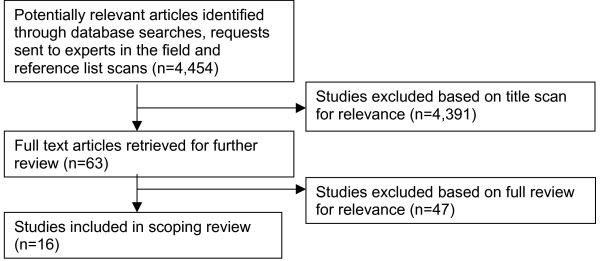
**Results of search strategy and process of selecting CAM related media research articles**. Figure 1 is a flow diagram describing the process of searching and selecting CAM related media articles to be included in the scoping review. Figure 1 is submitted as a separate Microsoft Word (2003) document.

Figure 1 outlines the results of the search strategy and study selection processes. Due to a lack of indexing of many CAM related terms, the search process identified a large number of irrelevant articles. Of the 4,454 articles identified through the various searches, 16 articles were selected for the final review [5-7,10,25-36].

Inter-rater reliability of the study selection and data extraction processes was high. In regards to study selection, there were 41 discrepancies representing 0.92% of the total. Each discrepancy was a case of one reviewer coding an article as "maybe" with the other coding it as "include" or "exclude". In all cases, the full article was retrieved and read by both reviewers to resolve the discrepancy. Inter-rater reliability of the descriptive data extraction process was 100% agreement.

### Descriptive summary of research on CAM and the mass media

#### Amount of research on CAM and the mass media

We located 16 articles that described research on CAM and mass media. All articles were published in 1998 or later with a maximum of three articles published in any year (see Additional file 2). Although four of the 16 articles (25%) were published in the last two years–2006 and 2007–there does not appear to be a growing trend towards publishing in this field in recent years.

#### Focus of research on CAM and the mass media

Newspapers and magazines were the most commonly researched media types. None of the articles addressed television as a media type, and only one analyzed radio programs.

The articles covered media from many countries, with some articles focusing on media from more than one country. Nine articles focused on media from the United Kingdom (UK), five from the United States (US) and four from Canada.

Most of the studies did not analyze media content related to a specific disease (n = 12); however, two studies focused on representations of CAM treatment for cancer, one focused on menopause, and one on Alzheimer's disease.

The majority of articles (n = 9) described media coverage of a range of CAM therapies, with some of these articles articulating differences within the range of therapies surveyed ("Differentiated" in Additional file 2) and others treating CAM in a unified manner ("Unified" in Additional file 2). Of those articles that focused on a particular CAM therapy, natural health products (NHPs) received the most attention with six articles taking NHPs (e.g., dietary supplements, herbal remedies) as their focus.

#### Nature of research on CAM and the mass media

Research in this field has been developed through a variety of disciplinary perspectives, most commonly pharmacy, complementary medicine, public health, sociology, nutrition and conventional medicine. The majority of articles were published as original research articles (n = 11) and the majority described content analyses (n = 11), with discourse analyses also being common (n = 4). One half of all articles we reviewed excluded a discussion of reliability in sampling and measurement, definitions for subjective outcomes or discussion of analytic approach (see Table 1). Media representation research was by far the most common. Each article discussed representation issues, but four also addressed some production issues and two had an additional focus on the reception of media content by audiences.

**Table 1:** Summary of reporting of indicators of research quality in research on CAM and the mass media*

Author, Year [refID]	Indicators of Research Quality not Reported in Research Article
Adelman, 2003 [25]	○ Excludes discussion of reliability in sampling and measurement
Bubela, 2006 [10]	○ Excludes discussion of reliability in sampling and measurement
Ernst and Schmidt, 2004 [28]	○ Excludes description of sampling procedure
	○ Excludes discussion of reliability in sampling and measurement
	○ Potential for bias as authors analyzing coverage of their own press release
Ernst and Weihmayr, 2000 [29]	○ Excludes discussion of reliability in sampling and measurement
	○ Excludes definition of subjective outcomes
Koper, 2006 [33]	○ Excludes description of sampling procedure
	○ Excludes discussion of reliability in sampling and measurement
	○ Excludes definition of subjective outcomes
Milazzo, 2006 [6]	○ Excludes discussion of reliability in sampling and measurement
	○ Time period sampled cannot show variation in reporting throughout year
	○ Excludes definition of subjective outcomes
Miles, 1998 [34]	○ Excludes description of approach to data analysis and verification
Reddy, 2000 [7]	○ Excludes description of methodological approach, e.g., sampling, data analysis, data verification

* following a broad assessment of standard quality criteria as adapted from Glasziou et al. [8], Creswell [41] and Morse & Richards [42].

### Summary of results published in CAM related media literature

In total, 246 single result statements were extracted, which we summarize below according to the three areas of media research: production, representation and reception. We developed several categories within the areas of media production and representation research as a means to simplify the presentation of topically similar results [24].

#### Production of CAM content

Issues related to the production of CAM related media information were addressed in four articles. Doel and Segrott [27] interviewed editors of British-based health and lifestyle magazines to determine how they perceive their audiences and correspondingly place CAM in those magazines. Other researchers examined the sources that journalists use to produce media texts [5,10,32,36].

##### Perceived audiences

Doel and Segrott [27] describe that the editors they interviewed perceive their audiences to be predominantly female, white, middle and upper class and committed to both healthy lifestyles and CAM. They perceived their role as editors to be the difficult task of translating the technical language of CAM into the accessible language of everyday consumerism. In many cases, editors aimed to help readers 'find out more', as a means to help them take responsibility for their health and to become informed and self-empowered.

##### Journalist sources

The number and type of sources that journalists rely on to produce CAM content appears to differ by country. In their review of CAM coverage in nine newspapers from five countries, Vastag et al. [36] found that an average of 4.4 sources were used per story, with newspapers based in the United States tending to use more sources than newspapers published in other countries included in their sample. Canadian newspaper and magazine coverage of CAM appears to most frequently (49.1%) cite only one source [5]. Vastag et al. [36] also report that conventional health care sources were cited more than twice as often as CAM sources, and for shorter articles (under 1,000 words) journalists cited conventional sources almost exclusively. Bubela et al. [10] also noted a tendency to cite conventional sources over CAM sources in their review of Canadian, US and UK-based media reports describing published herbal remedy clinical trials. They observed that only two of the clinical trials reported by the media (3% of their sample) were published in CAM journals and the remainder were published in conventional medical journals, such as the *British Medical Journal *and *The Lancet*. Finally, a reliance on personal anecdotes appears common in CAM related media reporting [5,26,32], as does lack of referencing published CAM research [26,32].

#### Representation of CAM content in the media

Representation issues were a focus of each article we reviewed. Accordingly, several categories emerged and are summarized below to provide a comprehensive view of how CAM is represented in the media.

##### CAM discourses and frames

Discourse can be defined as the way issues are commonly discussed, for example the language and rhetorical strategies used to make points [37]. Frames are related, but broader. They describe the structural nature of a text and may become apparent, for example, by examining what information is presented in an opening paragraph, what metaphors are used and what examples are provided. Frames are, in essence, an invitation to read a story in a particular way [38] and set the boundaries for discourse. CAM discourses and frames were the focus of five articles [7,26,27,32,34].

Carter's [26] analysis of US-based newspapers and women's magazines suggests CAM coverage employs conflict and controversy frames, with a debate between CAM and conventional medicine described in terms of credibility struggles between "scientifically sound" medicine on the one hand and unscientific alternatives on the other. Miles [34] and Reddy [7] similarly highlight the use of the scientific discourse, but as a strategy to suggest the legitimacy and credibility of CAM.

Doel and Segrott [27] uncovered three discourses in their research that share similar features to those presented by Kirkman [32], Miles [34] and Reddy [7]. In their first discourse–labelled "pragmatic toolkit"–CAM is portrayed as a collection of tools that may be used to cure illness or treat symptoms, without reference to the unique philosophical assumptions of many CAM therapies. Reddy [7] describes similar treatment of Ayurveda, with this coherent system of medical knowledge often being portrayed as a disarticulated set of self-help strategies. In the second discourse "from illness to healthy living", CAM is drawn into a broad notion of healthy living that encompasses almost every aspect of daily life [27]. CAM is portrayed as a means to cope with the "dis-ease" of urban life that may result from work-related stress and problems with relationships, time management and finances. Reddy [7] similarly describes that Ayurveda is often presented as the Eastern antidote to Western stresses of modernity and materialism. Miles [34] also uncovered a similar discourse specific to portrayals of herbal remedies and dietary supplements, but also notes a paradox in that these products purport to provide an alternative to the problems that capitalism has generated but the purchase of these products is required to do so, which supports the capitalism suggested to create the problems in the first place. In Doel and Segrott's [27] third discourse, CAM is placed at the heart of a "natural" or "alternative" lifestyle, disconnected from both the biomedical model of illness and the socially constructed dis-ease of everyday life, and alongside a concern with environmental, ecological and ethical issues.

##### Tone of coverage

As is suggested by most of these discourses, it appears that CAM coverage in the media is–for the most part–positive, although there may be some differences by country. Ernst and Weihmayr [29] suggest that the majority CAM reporting in both UK and German based newspapers is positive, although German reporting may be more critical. Vastag et al. [36] found coverage in five countries to be overwhelmingly positive, with 58% of the articles they reviewed containing some positive portrayal or promotion of CAM, while only 20% contained any negative portrayal. Similarly, for each of 16 years of Canadian newspaper and magazine coverage, Weeks et al. [5] judged a larger proportion of articles to be favourable towards CAM use for cancer than not (in total, 61.3% of magazine and 45.3% of newspaper articles were judged favourable).

##### Discussion of related risks and safety

Positive portrayals of CAM may be related to an under-representation of the potential risks associated with CAM use [10]. Only 23% of the articles reviewed by Weeks et al. [5] included a discussion of potential risks. Kava et al. [31] rated the quality of dietary supplement safety information presented in magazines popular among older readers. They found that the amount and quality of safety information varied greatly, with most articles presenting only partial information, if any. Only 16% of the 254 articles they reviewed for discussion of safety information received a rating of "excellent", 52% were rated "good" and 32% were rated "poor". Further, Carter's [26] review suggests that when potential risks of CAM use are presented to readers, they are presented in such a way that minimizes negative characterizations of CAM, thereby affording these cautions minimal attention.

##### Time trends

Six authors addressed trends over time in CAM reporting [5-7,10,31,35]. Although different time periods were considered and research foci varied, these studies suggest that the frequency of CAM coverage has increased over time in Canada [5], the UK [6] and the US [7], although it may have peaked in the mid-late 1990's in North America [5,7].

##### Differences between newspapers and magazines

The research reviewed in this sample highlights differences between newspaper and magazine reporting. For example, Weeks et al. [5] uncovered differences in regards to a focus on cancer type, tone of coverage, suggested reasons for CAM use, labels used to describe "CAM" and likelihood of providing various recommendations to readers. Gray et al. [30] suggest that newspapers cover CAM significantly more frequently than magazines and Carter [23] suggests that a described controversy over the safety and effectiveness of CAM treatments for menopause receives more attention in newspapers than magazines.

#### Reception of CAM related media content by audiences

The research we reviewed suggests that CAM related media content appeals to heterogeneous audiences through the use of discourses that resonate with various concerns of segments of the population at a given time. For example, the discourses used to present herbal remedies and dietary supplements to Ecuador's rural and marginalized urban residents resonate with their concerns regarding environmental degradation, tensions between traditionalism and modernism, frustrations about personal achievement and the place of Ecuador in the global marketplace [34]. Reddy [7] similarly suggests that different strategies of mediating CAM information appeal to different audiences. She describes five heterogeneous audiences that consume Ayurvedic information each in specific ways. For example, the non-intellectual American middle class form one audience that seeks information presented using a combination of scientific credibility and masculine rationality; and, a separate audience comprised of American and South Asian women relate to information that suggests the dual role of women as modern agents of change and holders of tradition.

## Discussion

There are several limitations to our scoping review that must be considered when interpreting our description and summary of research on CAM and the mass media. First, we located and summarized only 16 articles on the topic, which precludes a comprehensive assessment of such a diverse field. These 16 articles were rather diverse, which means we had to make decisions regarding which of the many meaningful results to summarize. In particular, for the qualitatively oriented research, summarizing only some results has thinned out the desired thickness of particular descriptions and has led to a loss of the complexity in the individual studies [16]. Further, due to time and cost considerations we included only English language publications. The exclusion of non-English language publications means that the results of our review should only be considered relevant for those countries whose media was analysed by researchers who published in English, primarily Canada, the United Kingdom and the United States. Although (some of) our results can be interpreted cautiously for countries such as New Zealand, Germany, Ecuador and Finland (that were included in our review), they may not be applicable to countries such as Italy, Russia, India or Brazil, for example. Also due to time and cost considerations, we made the decision to exclude research articles that described analysis of information available over the Internet. The Internet is increasingly becoming an important source of health information [20] but the results of our review cannot be generalized to this unique form of mass media. Finally a lack of indexing of relevant research by keywords we used in our search strategy may have led us to not locate and therefore summarize the results of some relevant research. For example, it is possible that some CAM related media reception research is embedded within published research indexed using information, decision-making, attitudes, beliefs and behaviour related keywords. For practical reasons, however, we were unable to exhaustively search all these potential bodies of literature. We do believe, though, that the majority of research with a media focus would have been indexed as such.

In light of these limitations in approach, it is possible to draw tentative conclusions bounded by the constraints of generalizability outlined above. First, it seems that CAM related media coverage has increased over the past decade, though has stabilized more recently in North America. Further, coverage is–for the most part–positive towards CAM, which may partially result from discursive strategies used to minimize attention to potential risks. Coverage is not, however, unanimously positive or without controversy. As with the traditional academic literature, a debate over the evidence-base of CAM is of central interest [39,40]. Finally, reporting attracts reader attention through several means, as CAM is constructed in different ways to appeal to diverse audience members through the use of familiar discourses.

Representing a variety of theoretical perspectives, the research reviewed here collectively supports the notion that increased media coverage of CAM is related to increased use of CAM in more recent years. There is quantitative evidence to support increased CAM coverage in more recent years and qualitative evidence to support the persuasive nature of that coverage for a variety of audiences. It is unclear, however, whether media information influences individual decisions to use CAM or the increased use of CAM by individuals provides an impetus for increased media coverage. It is likely a bit of both. What is clear is that the media remains an important source of CAM information for a variety of individuals and further research is needed on the reciprocal relationship between media coverage and CAM use.

This review is instructive for the future of this field, highlighting several important issues to guide future research. For example, there appears to be a need for many collaborative relationships. The research we reviewed spans multiple disciplines, but is not multi-disciplinary in the sense that theories and methods from multiple disciplines inform the research approach. In particular, research in this field could benefit from the use of a theoretical perspective that links together media production, representation and reception through its relation to culture. A unified perspective would help make sense of the diversity of research in this field, in particular by contextualizing the results through reference to the circuit of media communication (e.g., production → representation → reception → production, etc.). In addition to multi-disciplinary research relationships, collaborative relationships between journalists and researchers and researchers and CAM users should be established. Such relationships will help ensure research is informed by media practice as well as the context in which individuals use media information, and also that media practice is informed by research. Further, the differences we suggest are inherent between media published in different countries and between media types has important implications for future research sampling decisions. It also seems important that researchers recognize the many differences between the variety of products and therapies that are typically labelled "CAM". For example, it is reasonable to assume that different issues (e.g., representation of risks and benefits) may emerge as important related to media coverage of acupuncture or massage, as opposed to NHPs. Researching CAM as a unified concept is useful in the sense that CAM tends to represent that which is not in the culturally dominant position of biomedicine and therefore serves as the basis of resistance or opposition; however, as we learn more about the intricacies of this varied group of products and therapies researching CAM as a unified concept becomes less meaningful. We also recommend that researchers fully report their methodological approach, so that given differing conceptions of research quality research users can make their own judgements whether study results are useful to them.

Our review suggests unequal attention to the different areas of media research and different media types. Representation research was by far the most common, and it almost exclusively focused on the print media. The same focus has been observed with regards to conventional medicine related media research [11,37] and this is likely due to the ease with which data can be collected and analyzed for print media based representation research. Representation research alone cannot describe the relationship between media coverage and individuals or societies, however, as audience members do not simply accept everything they read or hear in the media and do not sample media content in the same ways as researchers do when designing study protocols. This critique should not be seen as criticism of research in this field, but instead a call to researchers to begin a more comprehensive, multi-disciplinary and theoretically informed exploration of the many and varied issues of concern.

## Conclusion

A scoping review of research on CAM and the mass media has identified a wide range of research and research approaches. However, despite the diversity in approach the review has highlighted some key concepts that should be considered when designing and reporting future research. Specifically, issues related to production and reception are in need of research attention, and focused studies that examine radio and television broadcasts are needed. Most importantly, however, future research needs to be collaborative, involving researchers across disciplines, journalists and CAM users so that issues related to production, representation and reception can be studied in a rigorous and comprehensive manner.

## Competing interests

The authors declare that they have no competing interests.

## Authors' contributions

LCW conceived of the study, designed the research protocol, conducted the literature search, participated in data collection and analysis and prepared a first draft of the manuscript. TS assisted with data collection, analysis and interpretation and assisted with drafting the manuscript. Both authors have read and approved the final manuscript.

## Pre-publication history

The pre-publication history for this paper can be accessed here:



## Supplementary Material

Additional File 1 **Search strategy used to identify CAM related media research articles**. outlines in table format the search strategy and results of the search strategy used in the scoping review. It includes search terms, databases searched and number of articles retrieved from each source. Click here for file

Additional File 2 **Descriptive summary of research on CAM and the mass media**. summarizes in table format various qualities of the research we reviewed on CAM and the mass media, including: author of research, year of research publication, author's home discipline, article type, study design, type of media, type of media research, country of media origin, and the disease and CAM focus of the reviewed research. Click here for file
