# Supplementary material for: A scoping review of research on complementary and alternative medicine (CAM) and the mass media: Looking back, moving forward
Source: BMC Complement Altern Med. 2008 Jul 19;8:43. doi: 10.1186/1472-6882-8-43 (PMC2494539; doi:10.1186/1472-6882-8-43)
Supplement: Additional File 1 — Search strategy used to identify CAM related media research articles. outlines in table format the search strategy and results of the search strategy used in the scoping review. It includes search terms, databases searched and number of articles retrieved from each source. [file 1472-6882-8-43-S1.docx]

## Table 1 - Search strategy used to identify CAM related media research articles

| Database searched, date searched | CAM/health care keywords or subject headings | Media keywords or subject headings | Number of References Retrieved |
| --- | --- | --- | --- |
| Communication Abstracts, Dec. 11, 2006 | Medical care; Health; Health care; Health communication; Health information; Public health | Mass media; News media; Print media; Broadcast media; Radio; Television; Mass communication; Magazines; Newspapers; News; Television news | 63 |
| Communication and Mass Media Complete, Dec. 11, 2006 | Communication in medicine; Journalism, medical | Local mass media; Mass media; Advertising; Marketing; Press; Mass media – research; Newspapers;, Periodicals; Prime time broadcasting; Radio; Radio broadcasting; Radio programs; Television; Television broadcasting; Television programs; Women’s magazines; Journalism | 57 |
| Cumulative Index to Nursing Allied Health Literature, Dec. 23, 2006 | Alternative therapies; Plants, medicinal; Dietary supplements; Drugs, Chinese herbal; Vitamins; Minerals | Radio; Television; Newspapers | 967 |
| EMBASE (1980-2006), Dec. 23, 2006 | Alternative medicine; Massage; Homeopathy; Manipulative medicine; Acupuncture; Ayurvedic drug; Plant extract; Traditional medicine; Medicinal plant; Yoga; Meditation; Vitamin; Mineral; Diet therapy | Television; Mass medium; Telecommunication; Publication | 1946 |
| MEDLINE (1966-2006), Dec. 23, 2006 | Complementary therapies; Plants, medicinal; Drugs, Chinese herbal; Plant extracts; Vitamins; Minerals; Dietary supplements | Radio; Television; Serial publications | 930 |
| Ovid Healthstar, Dec. 23, 2006 | Complementary therapies; Medicine, herbal; Plants, medicinal; Drugs, Chinese herbal; Phytotherapy; Dietary supplements; Vitamins | Mass media; Serial publications | 255 |
| AMED, Dec. 23, 2006 | Complementary medicine; Acupuncture; Chiropractic; Herbs; Plants medicinal; Herbal drugs; Dietary supplements; Plant extracts; Vitamins; Minerals; Diet therapy; Massage | Periodicals; Television; Publishing | 161 |
| SOCIndex, Dec. 11, 2006 | Alternative medicine; Holistic medicine; Traditional medicine; Self-care health; Acupuncture; Chiropractors; Meditation; Yoga; Herbs; Hypnotism – therapeutic use | Mass media; Newspapers; Periodicals; Radio; Television; Marketing; Radio broadcasting; Radio programs; Television broadcasting; Television programs; Serial publication;  Health in mass media | 32 |
| Social Sciences Abstracts, Dec. 11, 2006 | Alternative medicine; Acupuncture; Chiropractic; Holistic medicine; Homeopathy; Medicine, oriental; Naturopathy; Self care, health; Traditional medicine | Mass media; Health in mass media; Medical Care in mass media; Media use; Media culture and society; Newspapers; Periodicals; Physicians in mass media; Press; Public health in mass media; Radio broadcasting; Television broadcasting | 17 |
| Sociological Abstracts, Dec. 11, 2006 | Alternative medicine; Faith healing; Holistic medicine; Traditional medicine; Plants (botanical); Hypnosis; Meditation (transcendental); Yoga | Mass media; News media; Editorials; News coverage; Newspapers; Radio; Television; Periodicals; Magazines | 14 |
| Experts in the field |  |  | 4 |
| Reference list scan |  |  | 8 |
| Total References Retrieved |  |  | 4,454 |
