# Supplementary material for: A scoping review of research on complementary and alternative medicine (CAM) and the mass media: Looking back, moving forward
Source: BMC Complement Altern Med. 2008 Jul 19;8:43. doi: 10.1186/1472-6882-8-43 (PMC2494539; doi:10.1186/1472-6882-8-43)
Supplement: Additional File 2 — Descriptive summary of research on CAM and the mass media. summarizes in table format various qualities of the research we reviewed on CAM and the mass media, including: author of research, year of research publication, author's home discipline, article type, study design, type of media, type of media research, country of media origin, and the disease and CAM focus of the reviewed research. [file 1472-6882-8-43-S2.docx]

### Additional File 2 - Descriptive summary of research on CAM and the mass media

| Author, Year [refID] | Author’s home discipline(s) | Article type | Study Design | Type of media | Type of media research | Country of media origin | Disease focus | CAM focus |
| --- | --- | --- | --- | --- | --- | --- | --- | --- |
| Adelman 2003 [25] | Medicine | Letter to the editor | Content analysis | Newspapers | Representation | Canada, United Kingdom, United States | Alzheimer’s disease | Vitamin E |
| Bubela, 2006 [10] | Marketing, Business, Economics, Law, Pharmacy | Original research | Content analysis | Newspapers | Representation | Canada, United Kingdom, United States | None | Herbal remedies |
| Carter, 2001 [26] | Sociology | Dissertation | Discourse analysis | Newspapers, magazines | Representation | United States | Menopause | Unified CAM, Differentiated CAM |
| Doel and Segrott [27] | Geography | Original research | Discourse analysis | Magazines | Production, representation | United Kingdom | None | Unified CAM |
| Ernst and Schmidt, 2004 [28] | Complementary medicine | Editorial | Content analysis | Newspapers | Representation | United Kingdom | None | Unified CAM |
| Ernst and Weihmayr, 2000 [29] | Complementary medicine, medicine | Letter to the editor | Content analysis | Newspapers | Representation | Germany, United Kingdom | None | Unified CAM |
| Gray, 1998 [30] | Pharmacy | Original research | Content analysis | Newspapers, magazines | Representation | United Kingdom | None | Unified CAM, Differentiated CAM |
| Kava, 2002 [31] | Nutrition | Original research | Content analysis | Magazines | Representation | United States | None | Dietary supplements |
| Kirkman, 2001 [32] | Social and cultural studies | Original research | Discourse analysis | Magazines | Production, Representation | New Zealand | None | Unified CAM, Differentiated CAM |
| Koper, 2006 [33] | Law, Marketing, Economics, Business, Pharmacy | Original research | Content analysis | Newspapers | Representation | Canada, United Kingdom, United States | None | Herbal remedies |
| Milazzo, 2006 [6] | Complementary medicine | Original research | Content analysis | Newspapers | Representation | United Kingdom | Cancer | Unified |
| Miles,1998 [34] | Anthropology | Original research | Discourse analysis | Radio | Representation, reception | Ecuador | None | Herbal remedies, dietary supplements |
| Reddy, 2000 [7] | Sociology | Dissertation | Ethnography | Magazines | Representation, reception | United States | None | Ayurveda |
| Uusitalo, 2000 [35] | Public Health, Nutrition, Epidemiology, Health Promotion | Original research | Content analysis | Newspapers, magazines | Representation | Finland | None | Antioxidants |
| Vastag, 1999 [36] | Science and technology journalism | Original research | Content analysis | Newspapers | Production, representation | China, Israel, Japan, United Kingdom, United States | None | Unified CAM, Differentiated CAM |
| Weeks et al., 2007 [5] | Community health science, knowledge translation | Original research | Content analysis | Newspapers, magazines | Production, representation | Canada | Cancer | Unified CAM, Differentiated CAM |
